# Psychosis as a Possible Prodrome of Multiple Sclerosis: A Case Report

**DOI:** 10.1192/bjo.2022.76

**Published:** 2022-06-20

**Authors:** Mishal Abu Al-Melh, Mohammed Farghal, Nasser Abdelall

**Affiliations:** Adan Hospital, Kuwait, Kuwait

## Abstract

**Aims:**

Multiple sclerosis (MS) is an autoimmune disease characterized by focal demyelinating lesions that can affect any part of the central nervous system. The highlight of the disease is the wide range of neurological deficits; however, psychiatric manifestations are not uncommon. MS is associated with psychiatric comorbidities, such as depression and anxiety disorders. This is due to either its disease process or its therapies that have been well documented in the literature. However, the link between psychosis as a prodrome and MS remains understudied and relatively uncommon.

**Methods:**

We present a 40-year-old gentleman who developed isolated psychiatric manifestations for about 10 years in the form of progressive behavioral changes, social and occupational dysfunction, coupled with persecutory delusions. No personal or family history of mental illnesses. He was diagnosed and treated as a case of schizophrenia by a psychiatrist with multiple antipsychotics with a minimal improvement of his symptoms. Three months before our assessment the patient had a history of difficulty walking associated with urine and fecal incontinence. On examination, he had restricted affect and was easily agitated. Neurological examination revealed hypertonia and hyperreflexia in his left upper and lower limbs with normal power bilaterally. T2-weighted magnetic resonance imaging (MRI) brain showed multiple non-enhancing characteristic demyelinating lesions with typical shape and distribution. Further work-up was done to confirm the diagnosis of MS and exclude differentials, including a negative autoimmune screen, anti-aquaporin-4 (AQP4), and Myelin oligodendrocyte glycoprotein (MOG) antibodies. Visual evoked potentials documented bilateral severe disturbance in the visual pathway in both eyes, suggesting axonal loss. The patient is a candidate for a disease-modifying therapy for MS. Natalizumab or Ocrelizumab was selected based on his clinical criteria and will be started after proper preparation.

**Results:**

Psychiatric comorbidities in MS are associated with reduced compliance to disease-modifying therapies and lower quality of life. Studies reported that lesions in the periventricular area in the temporal region may be associated with psychosis, but not as an isolated presentation in patients with MS.

**Conclusion:**

MS is one of the differentials of psychotic disorder due to a medical condition, and rarely may present with pure psychiatric manifestations preceding any obvious neurological deficits, leading to delay in the diagnosis. Despite being uncommon, a high index of suspicion should be kept in patients with atypical presentations associated with limited response to multiple antipsychotics. This highlights the importance of conducting a thorough physical examination and work-up to exclude an organic pathology, necessitating proper management.

